# 2151: Using social network analysis to design and evaluate CTSA pilot programs

**DOI:** 10.1017/cts.2017.162

**Published:** 2018-05-10

**Authors:** Therese Kennelly Okraku, Valerio Leone Sciabolazza, Raffaele Vacca, Christopher McCarty

## Abstract

OBJECTIVES/SPECIFIC AIMS: We aim to leverage our analysis of the scientific collaboration network at a research university to design an innovative pilot program and foster scientific productivity. We test the impact of creating a new collaboration in a research community, which decreases the average network distance and accelerates the diffusion of information and expertise among the community’s investigators. METHODS/STUDY POPULATION: We mapped the whole network of co-authorship on publications and co-participation on extramurally awarded grants at the University of Florida (UF) between 2013 and 2015. We used network science methods to identify research communities of investigators who have consistently worked together and/or have other collaborators in common with at least one researcher based in the UF Health Science Center. We selected pairs of communities with (i) similar productivity levels, research interests, and network structures and (ii) no research projects in common. Communities in each pair were randomly assigned to a treatment or control group. In each treatment community, we selected 1 pair of investigators who had not collaborated in the past 3 years and whose connection would maximally reduce average network distance in the community. The pair was provided with an economic incentive to collaborate for the submission of a CTSA pilot proposal. RESULTS/ANTICIPATED RESULTS: We successfully identified 15 pairs of treatment/control communities. In each of 8 treatment communities, a pair of potential collaborators agreed to participate in the intervention. DISCUSSION/SIGNIFICANCE OF IMPACT: Network-informed Clinical Translational Science Awards (CTSA) pilot programs can identify research communities and create innovative collaborations. Statistical experiments can establish the programs’ causal effects on scientific productivity.

